# The Dangers of Premature Burial

**Published:** 1910-03-19

**Authors:** Jas. R. Williamson

**Affiliations:** 100 Chidington Road, Upper Edmonton, London, N.


### Editor's Letter-Box.

### THE DANGERS OF PREMATURE BURIAL.

*To the Editor of* The Hospital.

Sir,—Under our existing laws (which do not make it obligatory that an apparent corpse should be medically examined, and scientifically tested, in order to make sure that death has really supervened) premature burial, an able and instructive article on which eminently important subject was published in The Hospital of the 5th inst., is a constant peril to everyone who appears to die, except those killed outright by accident. It is by no means certain that all people are dead when buried. The narrow escapes from burial alive reported in the press from time to time, and the facts that come to the knowledge of the Association for the Prevention of Premature Burial from persons who, by the merest chance (humanly speaking), have avoided live burial, render it not only possible, but certain that some, if not many, have not the good fortune to escape, and are consigned to the grave whilst in a condition of suspended animation.

It is the usual practice in the medical profession to certify to the cause of death without any inspection of the body, and on the mere hearsay evidence that the patient is dead. When one contemplates the insuperable difficulties placed in the way of discovering premature burials, by reason of the rarity of exhumation (about one in 50,000 interments), that those who have escaped have done so, as it would seem, quite by accident, such as the twitching of an eyelid, lips, and other portions of the body, perspiration on the forehead, delay of a funeral, etc., and the constant burial of persons before putrefactive decomposition—the only really trustworthy proof of death—has set in, it cannot with reason be inferred that such ghastly tragedies do not occur in this country, nor that the dangers are infinitesimal and unworthy of consideration. Indeed, taking into consideration the facilities that exist, the great uncertainty of the so-called signs of death, the deceptive nature of forms of suspended animation, rightly termed death counterfeits, the fact that an annual average of 9,000 bodies are buried in England and Wales, and a larger proportion in Scotland and Ireland, without any sort of certificate, or any scientific proof of their decease, it would be very surprising if premature interment did not happen. Assuredly, before any thought of burial, cremation, post-mortem, or embalming is entertained, it should be obligatory to ascertain beyond the least shadow of doubt that the person is really, and not merely apparently, without life. This can only be ensured by applying a number of scientific tests, as shown by the late Sir Benjamin Ward Richardson, M.D., F.R.S., and in no case should be ever omitted.

In an address on premature burial before a medical society at Boston, U.S.A., the late Professor Alex. Wilder, M.D., concluded with the following impressive peroration: "The thought of suffocation in a coffin is more terrible than that of torture on the rack or burning at the stake. The fearful despair, however short the period, is too full of horror to contemplate with calmness. Carelessness in this matter cannot be innocent, indifference is culpable, and even ignorance in respect to it is closely akin to crime. Our sorrowing is a mockery, our tears little else than testimonials of our hypocrisy, when we neglect precautions against a fate so terrible—a fate to which everyone is more or less liable." If any of your readers are willing to help the humane movement for obtaining legislative precautions against the possibility of anyone being buried prematurely, I should be pleased to send literature on the subject on receiving a stamped addressed envelope. Thanking you for your kindness,

I am, Sir, your obedient servant,

Jas. R. Williamson.

100 Chidington Road, Upper Edmonton, London, N., March 8, 1910.

